# A Recombinant Lentiviral Vegfr2-Silencing Vector Attenuates Roxarsone-Promoted Growth of Rat Vascular Endothelial Cells and Angiogenesis in Matrigel Plug and B16F10 Xenograft Models

**DOI:** 10.3390/vetsci11100451

**Published:** 2024-09-24

**Authors:** Xin Chen, Lin Chen, Binlin Chen, Qianhan Wei, Yinchao Wu, Yumei Zhang

**Affiliations:** 1 Laboratory of Veterinary Pharmacology and Toxicology, College of Veterinary Medicine, Yangzhou University, Yangzhou 225009, China; xinchen@yzu.edu.cn (X.C.);; 2 Jiangsu Co-Innovation Center for Prevention and Control of Important Animal Infectious Diseases and Zoonoses, Yangzhou 225009, China

**Keywords:** roxarsone, VEGFR2, angiogenesis, vascular endothelial cells, matrigel plug, B16F10 xenograft

## Abstract

**Simple Summary:**

Roxarsone, a feed addictive to promote animal growth, has been found to induce angiogenesis. This study aimed to investigate the effect of a recombinant lentiviral Vegfr2-silencing vector on angiogenesis and carcinogenesis by roxarsone. The results showed that silencing the Vegfr2 gene by RNA interference through lentiviral vector attenuated roxarsone-induced growth of rat vascular endothelial cells and angiogenesis in matrigel plugs and B16F10 xenografts. The finding from this work provides a promising approach in the prevention and treatment of angiogenesis and tumorigenesis promoted by roxarsone.

**Abstract:**

Roxarsone (ROX) is widely used as a feed addictive for livestock and poultry. ROX promotes angiogenesis, which can lead to health problems, and it is necessary to identify methods to counter this angiogenic effect of ROX. The VEGF/VEGFR2 signaling pathway is involved in the growth and reconstruction of new blood vessels during angiogenesis. In this study, a recombinant lentiviral vector encoding Vegfr2 shRNA was transfected into rat vascular endothelial cells and used in mouse matrigel plug and melanoma xenograft models to investigate its potential to regulate ROX-induced angiogenesis and tumor growth. Treating endothelial cells with ROX increased cell proliferation, migration, and a tube-like structure of growth relative to the control group. The addition of the lentiviral Vegfr2-silencing vector significantly attenuated the effects of ROX on endothelial cells. The hemoglobin content of mouse matrigel plugs treated with ROX was increased significantly. This effect was dramatically attenuated by the co-administration of shRNA targeting Vegfr2. The volume, weight and CD34 staining of the melanoma xenograft tumors increased by ROX were also attenuated by Vegfr2 silence. These results indicate that the down-regulation of VEGFR2 protein plays an inhibitory role in the ROX-promoted angiogenesis in vivo and in vitro. These data support the targeting of Vegfr2 gene as an effective means to treat ROX-induced angiogenesis and tumor growth.

## 1. Introduction

Roxarsone (3-nitro-4-hydroxyphenylarsenic acid, ROX) is an organic arsenic compound which is often used as a feed additive to promote growth, improve immunity and pigmentation, and prevent selenium poisoning [1,2]. Aromatic organic arsenic additives, such as ROX, have been banned by many developed countries but are still widely used in many developing countries [3,4]. Previously, we found that ROX induces angiogenesis in vitro, ex vivo and in vivo [5,6,7]. ROX is less absorbed in animals, and most of them will enter the natural environment with feces, which brings a risk of environmental arsenic pollution [8]. Arsenic has been listed as a Class A carcinogen by the International Agency for Research on Cancer (IARC), and the environmental pollution caused by ROX used in animal production poses an environmental carcinogenic risk [9]. Exposure to different forms of arsenic can cause serious health problems, including cancer, neuronal disease, skin damage and cardiovascular disease [10,11].

Angiogenesis is markedly perturbed in the initiation of carcinogenesis and tumor progression, and the proliferation and growth of vascular endothelial cells are the basis of angiogenesis in the body [12]. Vascular endothelial growth factor (VEGF) is the most powerful angiogenic factor known to be produced by tumor cells [13]. VEGF must specifically bind with its receptor (VEGFR) for the biological effects to appear, including increased vascular permeability, increased angiogenesis, enhanced mitosis, proliferation and migration of tumor cells, and degeneration of extracellular matrix [14]. VEGFR2 is the primary VEGF receptor involved in angiogenesis and mitosis, and the VEGF/VEGFR2 signal pathway is currently the main target of VEGF-targeted antitumor therapy [15].

RNA interference (RNAi) is an effective means of inhibiting gene expression [16]. Short hairpin RNAs (shRNAs) are synthetic RNA molecules that mediate RNAi and require plasmid or viral/bacterial vector systems for expression [17]. Lentiviral vector is a replication-defective virus remodeled by HIV-1, which is an ideal vector for expressing shRNA [18]. The use of recombinant lentiviral vectors to carry and deliver shRNA has many advantages and is often used for germline delivery and the creation of in vivo animal models [19].

Here, we transduced rat vascular endothelial cells with a recombinant lentiviral vector encoding Vegfr2-targetedshRNA. We also used Vegfr2-silencing constructs in a mouse matrigel plug model in vivo, and in a mouse melanoma xenograft model, to investigate the regulatory potential of Vegfr2 silencing to counteract ROX-induced angiogenesis and tumor growth. Administration of Vegfr2-targetedshRNA significantly attenuated the ROX-induced growth of rat vascular endothelial cells and angiogenesis in Matrigel plugs and B16F10 transplanted tumors. These data indicate that silencing the Vegfr2 gene by RNA interference through lentiviral vector may be a promising strategy for the treatment of ROX-promoted angiogenesis and tumorigenesis.

## 2. Materials and Methods

### 2.1. Reagents

Roxarsone was purchased from TCI Chemical Trading Co., Ltd. (Shanghai, China). Rabbit anti-rat VEGFR2 polyclonal antibody, mouse anti-rat β-actin monoclonal antibody, HRP-labeled goat anti-rabbit IgG, HRP-labeled goat anti-mouse IgG and SABC-POD three-step detection kit were purchased from Boster Biological Technology Co., Ltd. (Wuhan, China). Rabbit anti-mouse CD34 polyclonal antibody was purchased from Beyotime Biotechnology Co., Ltd. (Shanghai, China). Hemoglobin determination kit was purchased from Jiancheng Bioengineering Research Institute (Nanjing, China).

### 2.2. Animals and Cells

All animal protocols were approved by the Committee for the Ethics of Animal Experiments of Yangzhou University. Wistar rats (110~150 g), male ICR mice and C57BL/6 mice (20 ± 2 g) were purchased from the Center of Comparative Medicine, Yangzhou University. The animals were raised under normal conditions. C57BL/6 mice were fasted for 12 h before gavage.

The thoracic aorta of Wistar rats was separated and cut into 1 mm^3^ tissue blocks; the blocks were implanted in a gelatin-covered cell bottle, ensuring that the inner membrane was in contact with the gelatin. In total,1 mL of Dulbecco’s modified Eagle’s medium (DMEM) was added and supplemented with 10% fetal bovine serum (FBS) to cover the block, then cultivated in a 37 °C, 5% CO_2_ constant temperature incubator. After 6 days, when the tentacle-shaped endothelial cells migrated out and had grown well, the plant block was discarded and the cultivation was continued with complete medium. The isolated endothelial cells were cultured with DMEM containing 10% FBS and 5 ng/mL VEGF. The third-generation vascular endothelial cells were collected for the following experiments.

Melanoma B16F10 cell line was donated by the Research Group of Laboratory Animals (College of Veterinary Medicine, Yangzhou University). The cells were maintained in DMEM with 10% FBS.

### 2.3. Recombinant Lentivirus and Infection of Cells

Recombinant lentiviral vectors encoding rat Vegfr2 or LV3 (for negative control) shRNA were generated. The shRNA-targeting-Vegfr2 sequence is as follows: 5′-CCGAATCCCTGTGAAGTAT-3′; the sequence for LV3-NC is as follows: 5′-TTCTCCGAACGTGTCACGT-3′. After infecting cells with 40 multiplicity-of-infection (MOI) recombinant lentiviruses and 0.5 μg/mL polybrene, the virus titer can reach 1 × 10^9^ TU/mL, and the infection efficiency can reach more than 90%.

### 2.4. Western Blot Analysis

The experiment is divided into 5 groups: blank control group (PBS group), infection negative control group (NC group), 1.0 μM ROX treatment group (ROX group), shRNA interference-targeting VEGFR2 group (sh-Vegfr2 group) and shRNA interference and ROX treatment group (sh-Vegfr2 + ROX group). Endothelial cells were transfected with recombinant lentiviruses for transfection experiments. After transfection, cells were cultured with the final concentration of 1.0 μM ROX for 24 h. Then, the cells were collected to extract the protein for Western blot analysis of VEGFR2.

### 2.5. BrdU Proliferation Test

The endothelial cells were transfected with/without lentivirus and/or treated with/without ROX, then 30 μM BrdU was added for 3 h. The cells were fixed with 4% paraformaldehyde for 30 min and permeabilized with 0.1% Triton X-100 for 15 min. After being treated with 2M HCl for 30 min, 0.1 M boric acid was added for neutralization for 10 min. In total, 5% BSA dropwise was added to the seal at room temperature for 30 min to block nonspecific binding. Then, 1:200 diluted BrdU primary antibody was added dropwise and incubated overnight at 4 °C. The next day, the primary antibody was discarded and FITC-labeled secondary antibody was added (diluted 1:50) dropwise under dark conditions. The antibody was then incubated for 1 h at room temperature and then 0.3 mL of DAPI staining solution was added at room temperature. After incubating for 3 min in the dark, the cells were observed under a fluorescence microscope. The nuclei that emit green fluorescence were marked as BrdU-positive cells. The nucleus stained with DAPI under the same field of view was blue. For each treatment, 5 fields of view were randomly selected to take pictures and counted. The cell proliferation ability was evaluated by the ratio of the number of BrdU-positive cells to the number of DAPI-positive cells.

### 2.6. Scratch Test

A sterile 200 μL pipette tip was used to mark a blank area on single-layer endothelial cells which were infected by lentiviruses vertically along a sterile steel ruler, then the marked endothelial cells were washed off with PBS and placed behind to cultivate in a 37 °C, 5% CO_2_ incubator. The sample was then observed and photographed under an inverted microscope at 0 and 24 h, respectively. ImageJ software was used to analyze the width (pixels) between cells on both sides of the scratch, and the difference between the scratch width at 0 and 24 h was used as the migration distance of vascular endothelial cells to evaluate the migration ability of endothelial cells.

### 2.7. Matrigel Tubule Formation Test

The matrigel, 96-well plate and pipette tip were precooled at 4 °C overnight. On the ice box the next day, 50 μL matrigel was added to each well and cured at 37 °C for half an hour. Then, the cells infected with lentivirus were added to each well, and 1.0 μM ROX was additionally added to the ROX group, incubated in a 37 °C incubator for 4 h, and then observed and photographed under an inverted microscope. The angiogenesis analysis plug-in of ImageJ was used to analyze the tube formation indicators of the vascular-like structure, and the number of angiogenesis nodes and the length of the vascular branch were used as indicators to evaluate the formation of the tube-like structure.

### 2.8. Hemoglobin Amount Determination in Mouse Matrigel Plugs

The PBS group and ROX group were normal rat vascular endothelial cells, and the sh-Vegfr2 + ROX group, sh-Vegfr2 group and NC group were all cells transfected with recombinant lentivirus. The ROX working solution was added to the cells of ROX group and sh-Vegfr2 + ROX group to a final concentration of 1.0 μM, and an equal volume of PBS solution was added to PBS group, sh-Vegfr2 group and NC group. The cell suspension of each group was evenly mixed with matrigel, heparin solution and VEGF solution, and injected into the back of ICR mice subcutaneously to establish a mouse matrigel plug model. After two weeks, the mice were sacrificed and the matrigel plugs that had been formed were peeled off. In total, 1 mL PBS solution was added to the plugs, which were cut into pieces to make a tissue homogenate. The homogenate was centrifuged at 12,000 r/min for 10 min and then the supernatant was taken. In total, 0.01 mL of the supernatant was mixed with 2.5 mL of the developer and let to stand for 5 min. The OD value of each tube was measured at 540 nm and 1 cm optical path. Free hemoglobin (g/L) = 367.7 × (determined sample OD value − blank OD value).

### 2.9. Growth and Angiogenesis Evaluation of Mouse B16F10 Xenograft Tumor

B16F10 cell infections with or without recombinant lentiviruses were subcutaneously injected into the right armpit of C57BL/6 mice to establish a mouse melanoma model. The ROX group and the sh-Vegfr2 + ROX group were given 25 mg/kg ROX via gavage every day, and the other groups were given PBS via gavage every day as a control for 7 days. Approximately 4 days after the injection, protrusions were seen at the injection site. The long diameter (L) and short diameter (W) of the transplanted tumor were measured with a vernier caliper, and the volume was calculated as V = 1/2 × L × W^2^. After another week, a necropsy was performed, the tumor was peeled off, weighed, and slices were made. Immunohistochemical staining for CD34 was performed to observe the tumor tissue angiogenesis. Briefly, tumor slices underwent deparaffinization, followed by inactivation of endogenous peroxidase with 3% peroxide for 10 min. Antigen retrieval was then carried out in 0.1 M sodium citrate using a microwave oven. Subsequently, the sections were blocked with 5% BSA and incubated overnight at 4 °C with polyclonal antibodies against CD34 (diluted 1:200). The samples were then stained using an SABC-POD three-step detection kit as per the manufacturer’s guidelines and visualized under a microscope.

### 2.10. Statistical Analysis

GraphPad Prism software (Version 5.01)was used for data processing, and the results were all expressed as mean ± standard deviation (mean ± SD). One-way analysis of variance (ANOVA) followed by Dunnett’s post hoc test or two-way ANOVA followed by Bonferroni’s post hoc test for multiple comparisons was used for significance analysis; *p* < 0.05 is considered statistically significant.

## 3. Results

### 3.1. Effect of Recombinant Lentivirus Targeting Vegfr2 on the Expression of VEGFR2 Protein in Rat Vascular Endothelial Cells Exposed to ROX

**Figure 1:**
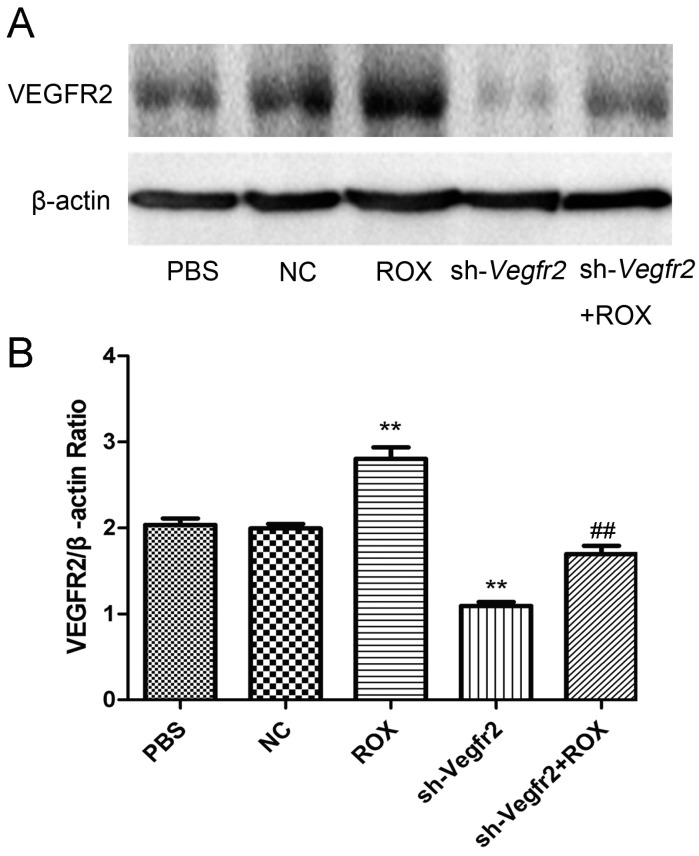
Effects of ROX and/or sh-Vegfr2 on VEGFR2 protein expression in rat vascular endothelial cells. (**A**) Western blot bands of indicating proteins in endothelial cells; (**B**) densitometric analysis of the ratio of the intensity of protein bands. Values are the mean ± SD of VEGFR2 expression standardized to β-actin expression in three independent experiments; ** *p* < 0.01, compared with the PBS group; ## *p* < 0.01, compared with the ROX group.

Figure 1 shows the expression of VEGFR2 in endothelial cells after different treatments. The Western blot bands of VEGFR2 protein in vascular endothelial cells are shown in Figure 1A. Figure 1B shows the analysis and statistics of the gray value of the protein bands. Compared with the blank control (PBS) group, negative shRNA (NC) had no significant effect on the expression of VEGFR2 protein (*p* > 0.05), while the protein expression level was reduced extremely in the sh-Vegfr2 group (*p* < 0.01), which indicates that the VEGFR2 signaling pathway was down-regulated successfully in endothelial cells. Compared with the PBS group, the expression of VEGFR2 protein was increased significantly in the 1.0 μM ROX treatment group (*p* < 0.01), while compared with the ROX group, the VEGFR2 protein level was decreased remarkably in the sh-Vegfr2 + ROX group (*p* < 0.01). The original images of the Western blot are published as supplementary material.

### 3.2. Effects of Recombinant Lentivirus Targeting Vegfr2 on the Growth of Endothelial Cells Promoted by ROX

#### 3.2.1. Effect on the Proliferation of Endothelial Cells

**Figure 2:**
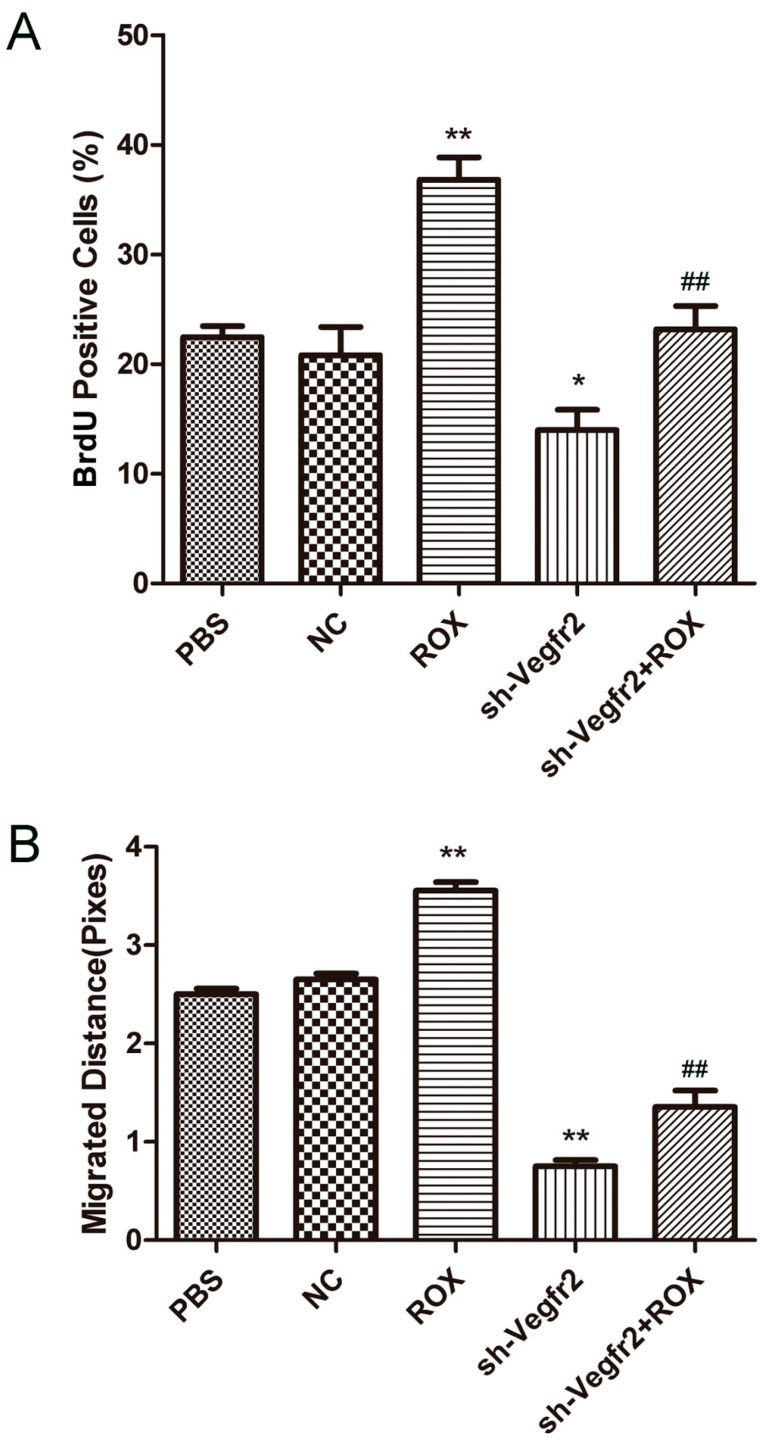
Effects of ROX and/or sh-Vegfr2 on proliferation and migration of endothelial cells. (**A**) BrdU-positive cell rate; (**B**) migration distance statistics chart. Data expressed as mean ± SD; * *p* < 0.05, ** *p* < 0.01, compared with the PBS group; ## *p* < 0.01, compared with the ROX group.

The statistical result is the ratio of cells stained with BrdU-FITC to cells stained with DAPI as the proliferation rate of endothelial cells (Figure 2A). Compared with the PBS group, 1.0 μM ROX dramatically increased the proliferation rate of endothelial cells (*p* < 0.01), while the cell proliferation rate was decreased significantly in the sh-Vegfr2 group (*p* < 0.05). Compared with the ROX group, the endothelial cell proliferation rate was reduced extremely in the sh-Vegfr2 + ROX group (*p* < 0.01).

#### 3.2.2. Effect on the Migration of Endothelial Cells

As shown in Figure 2B, both the ROX group and sh-Vegfr2 group have an effect on endothelial cell migration. Compared with the PBS group, the migration ability of endothelial cells was significantly enhanced in the ROX group (*p* < 0.01), while the cell migration ability was extremely reduced in the sh-Vegfr2 group (*p* < 0.01). Compared with the ROX group, the endothelial cell migration ability was remarkably reduced in the sh-Vegfr2 + ROX group (*p* < 0.01).

#### 3.2.3. Effect on the Ability of Endothelial Cells to Form In Vitro Tubes

**Figure 3:**
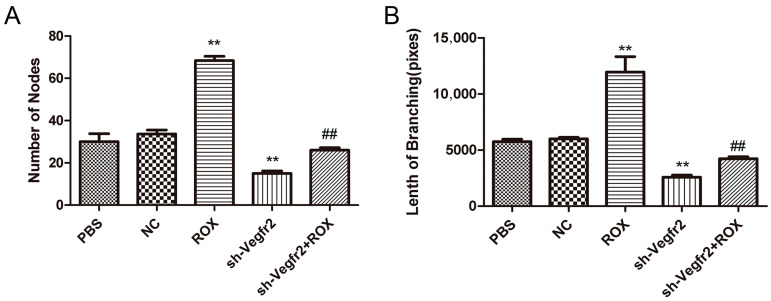
Effects of ROX and/or sh-Vegfr2 on tube formation of endothelial cells. (**A**) Number of nodes forming a tube-like structure; (**B**) branch lengths forming the tube-like structure. Data expressed as mean ± SD; ** *p* < 0.01, compared with the PBS group; ## *p* < 0.01, compared with the ROX group.

Different treatments also have a great impact on the endothelial cell tube formation ability (Figure 3), and the results are consistent with the results of the effects on the proliferation and migration of endothelial cells. Compared with the PBS group, 1.0 μM ROX significantly increased the number of nodes and branch length of the tube-like structure (*p* < 0.01), and the number of nodes and branch length were significantly decreased in the sh-Vegfr2 group (*p* < 0.01). Compared with the ROX group, the number of nodes and branch length were reduced dramatically in the sh-Vegfr2 + ROX group (*p* < 0.01).

### 3.3. Effects of Recombinant Lentivirus on ROX-Promoted Growth of Mouse Matrigel Plugs

#### 3.3.1. Establishment of Mouse Matrigel Plug Model

**Figure 4:**
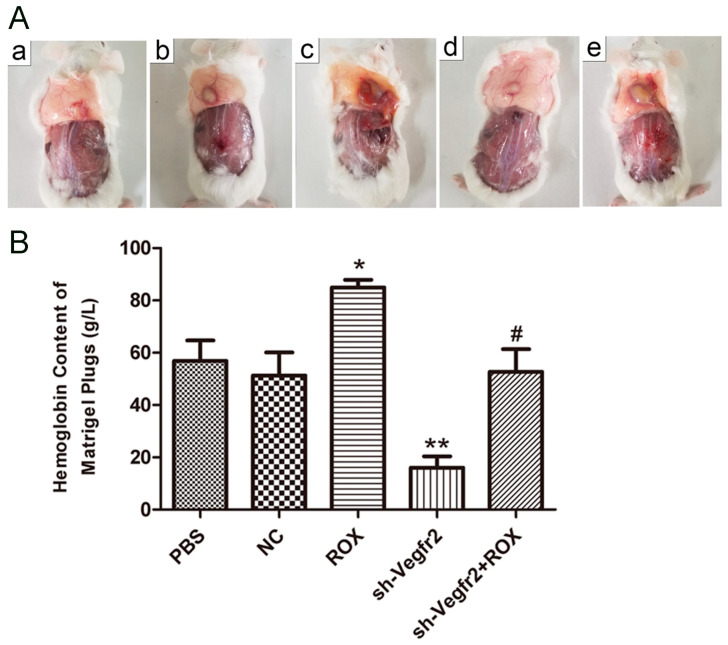
Effects of ROX and/or sh-Vegfr2 on the mouse matrigel plug model. (**A**) Pictures of the mouse matrigel plugs (a: PBS group, b: NC group, c: ROX group, d: sh-Vegfr2 group, and e: sh-Vegfr2 + ROX group); (**B**) hemoglobin content in matrigel plugs. Data expressed as mean ± SD; * *p* < 0.05, ** *p* < 0.01, compared with the PBS group; # *p* < 0.05, compared with the ROX group.

Obvious protrusions can be observed on the back of the mice in about 10 days, and the fingers are round bulges. As shown in Figure 4A, from the perspective of the size of the rubber plugs formed, the rubber plugs in the ROX group are larger than the PBS group, and those in the sh-Vegfr2 + ROX group are smaller than the ROX group; the rubber plugs are the darkest in the ROX group, followed by the sh-Vegfr2 + ROX group.

#### 3.3.2. Influence on the Content of Hemoglobin in Matrigel Plugs

It can be seen from Figure 4B that the hemoglobin content was the highest in the ROX group, which was significantly higher than the PBS group (about 1.49 times) (*p* < 0.05). The hemoglobin content in the sh-Vegfr2 group was extremely lower than the PBS group (about 0.28 times) (*p* < 0.01). The hemoglobin content in the sh-Vegfr2 + ROX group was significantly lower than that in the ROX group (*p* < 0.05), but there was no significant difference from the PBS group (*p* > 0.05).

### 3.4. Effects of Recombinant Lentivirus on ROX-Promoted Growth of Mouse Melanoma Xenografts

#### 3.4.1. Tumor Growth Evaluation

**Figure 5:**
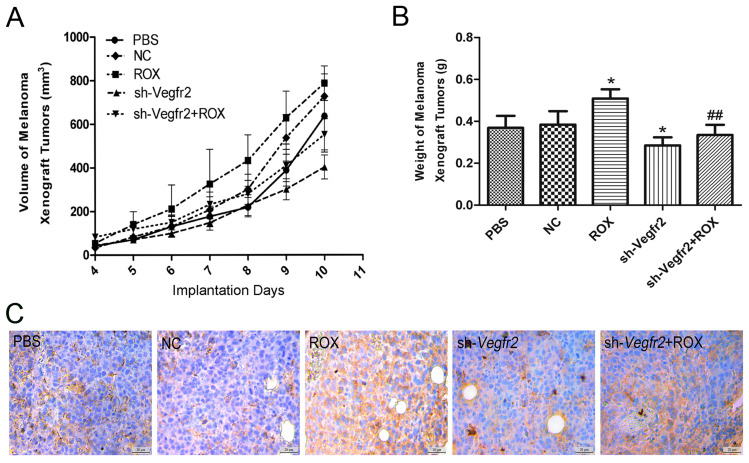
Effects of ROX and/or sh-Vegfr2 on the growth and angiogenesis of/in melanoma xenografts. (**A**) Melanoma xenograft tumor volumes during the test period; (**B**) melanoma xenograft tumor weight at necropsy; (**C**) immunohistochemical expression of CD34 in melanoma xenografts. Data expressed as mean ± SD; * *p* < 0.05, compared with the PBS group; ## *p* < 0.01, compared with the ROX group.

As shown in Figure 5A, the tumor volumes of the sh-Vegfr2 + ROX group were smaller in size than those of the ROX group on the 10th day. Compared with the PBS group, ROX significantly increased the weight of transplanted tumors (*p* < 0.05), and the weight was decreased significantly in the sh-Vegfr2 group (*p* < 0.05). Compared with the ROX group, the weight of transplanted tumors was reduced significantly in the sh-Vegfr2 + ROX group (*p* < 0.01) (Figure 5B).

#### 3.4.2. Immunohistochemistry of Tumor Tissue

The CD34 immunohistochemical results of tumor paraffin sections after different treatments can be seen in Figure 5C. CD34-positive reactions were yellow, and were mainly distributed in the cytoplasm and membrane of vascular endothelial cells. In the PBS group, a CD34-positive expression was seen in some capillaries. Compared with the PBS group, the expression of CD34 was reduced relatively in the sh-Vegfr2 group, while the expression of CD34 was increased in the ROX group. Importantly, the expression of CD34 in the sh-Vegfr2 + ROX group was less than that in the ROX group.

## 4. Discussion

The development and metastasis of tumors are closely related to angiogenesis [20]. Therapies targeting angiogenesis are of great interest in modern tumor treatment research [21,22,23]. Studies have found that the VEGF/VEGFR2 signaling pathway is the most important rate-limiting step in physiological and pathological angiogenesis [24]. As an emerging RNA interference technology, lentiviral expression vector provides a reliable and stable means for tumor treatment [25]. In previous studies, we showed that ROX increases the activity of endothelial cells, up-regulates the expression of VEGFR2 in endothelial cells, and promotes the growth and angiogenesis of mouse matrigel plugs and mouse melanoma xenografts [26,27]. This study used a recombinant lentiviral vector to down-regulate VEGFR2 in vitro experiments on endothelial cells and in vivo experiments on matrigel plugs and transplanted tumors to verify its therapeutic effect on ROX-induced angiogenesis.

Firstly, Figure 1 shows that the expression of VEGFR2 in the recombinant lentiviral vector infection group (sh-Vegfr2 group) was lower than that in the control group (PBS group), suggesting that the silence of Vegfr2 was successful in endothelial cells. Importantly, ROX-increased expression of VEGFR2 protein was significantly decreased by using the sh-Vegfr2 recombinant lentiviral vector. It means that the administration of recombinant lentiviral targeting Vegfr2 can down-regulate ROX-induced VEGFR2 signaling in endothelial cells.

The process of in vitro angiogenesis is mainly divided into cell proliferation, basement membrane degradation, and then proliferated cell migration to form new blood vessels [28]. As the initial link of angiogenesis, cell proliferation is the basis of angiogenesis [29]. Cell migration is the key to the process of angiogenesis, which determines the direction of blood vessel formation [30]. Vascular endothelial cell proliferation can be regulated by VEGF signaling [31,32]. In this study, the BrdU staining results showed that 1.0 μM ROX could significantly promote the proliferation of endothelial cells, and the rate of new cells in the ROX and Vegfr2 shRNA co-treatment group was significantly lower than that of the ROX group (Figure 2A). Figure 2B shows that 1.0 μM ROX could significantly promote the migration of endothelial cells, and co-treatment with the shRNA-targeting Vegfr2 gene significantly attenuated the promoted migration ability of endothelial cells by ROX. In the tube formation experiment, the results of the study are consistent with the results of the previous two experiments (Figure 3). The number of nodes and the length of tubules formed in the 1.0 μM ROX group were significantly increased compared to the PBS group, and the two indicators in the ROX + Vegfr2 shRNA group were significantly reduced. These show that VEGFR2-related signaling pathways have an important impact on the proliferation, migration and tube formation ability of endothelial cells induced by ROX.

Matrigel, an extract of a tumor containing all of the components present in basement membrane degradation and very biologically active, is widely used for studies on cell differentiation, angiogenesis, and tumor growth [33].The matrigel plug model is one of the most used assays for angiogenesis in vivo [34].Cancer mouse models have consistently served as a reliable platform for evaluating the efficacy of novel anti-cancer therapies. Among these models, xenografts of tumors implanted subcutaneously into mice have emerged as the most frequently utilized [35]. In the matrigel plug and the mouse B16F10 melanoma xenograft models, ROX can significantly promote an angiogenetic effect, and shRNA targeting Vegfr2 can inhibit the promotion of ROX (Figure 4 and Figure 5). This indicates that the VEGFR2 pathway has a regulatory role in the ROX-promoted growth of blood vessels. Down-regulating VEGFR2 expression through using a recombinant lentiviral vector is an efficient method for the treatment of angiogenesis and carcinogenesis promoted by ROX.

## 5. Conclusions

In the present study, shRNA targeting the Vegfr2 gene has a significant inhibitory effect on the growth of endothelial cells in vitro, and can effectively inhibit the ROX-induced angiogenesis of matrigel plugs and melanoma xenografts in vivo. Down-regulation of VEGFR2 protein plays an important regulatory role in ROX promoting angiogenesis in vivo and in vitro. It suggests that administration of shRNA targeting the Vegfr2 gene may be a potential approach in ROX-induced angiogenesis therapy.

## Data Availability

The original contributions presented in the study are included in the article/supplementary material, further inquiries can be directed to the corresponding author/s.

## Supplementary Materials

The following are available online at https://www.mdpi.com/article/10.3390/vetsci11100451/s1, Figure S1: Western blot image for VEGFR2.

## References

[1] Johnston N.L., Quarles C.L., Fagerberg D.J. (1983). Long-term broiler performance with bambermycins and bambermycins plus roxarsone. Poult. Sci..

[2] Hendrick C., Klug H.L., Olson O.E. (1953). Effect of 3-nitro-4-hydroxyphenylarsonic acid and arsanilic acid on selenium poisoning in the rat. J. Nutr..

[3] Nascimento A.L.A., Figueiredo I.M., Botero W.G., Santos J.C.C. (2023). Interaction between roxarsone, an organic arsenic compound, with humic substances in the soil simulating environmental conditions. Chemosphere.

[4] Mangalgiri K.P., Adak A., Blaney L. (2015). Organoarsenicals in poultry litter: Detection, fate, and toxicity. Environ. Int..

[5] Chen X., Zhang M., Chen L., Zhou Z., Chen B., Wang C., Xie Y., Zhang Y. (2021). Roxarsone Promotes Glycolysis and Angiogenesis by Inducing Hypoxia-Inducible Factor-1alpha In Vitro and In Vivo. ACS Omega.

[6] Zhang Y., Wang Y., Lu Q., Xin W., Cui W., Zhu J. (2016). Organoarsenic Roxarsone Promotes Angiogenesis In Vivo. Basic Clin. Pharmacol. Toxicol..

[7] Zhu J., Cui W., Liu X., Ying J., Hu C., Zhang Y. (2013). In vitro and ex vivo angiogenic effects of roxarsone on rat endothelial cells. Toxicol. Lett..

[8] Liu Y., Tian X., Cao S., Li Y., Dong H. (2021). Pollution characteristics and health risk assessment of arsenic transformed from feed additive organoarsenicals around chicken farms on the North China Plain. Chemosphere.

[9] Bhattacharjee P., Das A., Giri A.K. (2020). Epigenetic regulations in alternative telomere lengthening: Understanding the mechanistic insight in arsenic-induced skin cancer patients. Sci. Total Environ..

[10] Abdul K.S., Jayasinghe S.S., Chandana E.P., Jayasumana C., De Silva P.M. (2015). Arsenic and human health effects: A review. Environ. Toxicol. Pharmacol..

[11] Hughes M.F., Beck B.D., Chen Y., Lewis A.S., Thomas D.J. (2011). Arsenic exposure and toxicology: A historical perspective. Toxicol. Sci. Off. J. Soc. Toxicol..

[12] Kaneda H., Arao T., Matsumoto K., De Velasco M.A., Tamura D., Aomatsu K., Kudo K., Sakai K., Nagai T., Fujita Y. (2011). Activin A inhibits vascular endothelial cell growth and suppresses tumour angiogenesis in gastric cancer. Br. J. Cancer.

[13] Ferrara N., Adamis A.P. (2016). Ten years of anti-vascular endothelial growth factor therapy. Nat. Rev. Drug Discov..

[14] Ferrara N. (2004). Vascular endothelial growth factor: Basic science and clinical progress. Endocr. Rev..

[15] Shibuya M. (2006). Differential roles of vascular endothelial growth factor receptor-1 and receptor-2 in angiogenesis. J. Biochem. Mol. Biol..

[16] Yu J.Y., DeRuiter S.L., Turner D.L. (2002). RNA interference by expression of short-interfering RNAs and hairpin RNAs in mammalian cells. Proc. Natl. Acad. Sci. USA.

[17] Sui G., Soohoo C., Affarel B., Gay F., Shi Y., Forrester W.C. (2002). A DNA vector-based RNAi technology to suppress gene expression in mammalian cells. Proc. Natl. Acad. Sci. USA.

[18] Nishitsuji H., Kohara M., Kannagi M., Masuda T. (2006). Effective suppression of human immunodeficiency virus type 1 through a combination of short- or long-hairpin RNAs targeting essential sequences for retroviral integration. J. Virol..

[19] Grimm D., Kay M.A. (2007). RNAi and gene therapy: A mutual attraction. Hematol. Am. Soc. Hematol. Educ. Program.

[20] Claesson-Welsh L., Welsh M. (2013). VEGFA and tumour angiogenesis. J. Intern. Med..

[21] Li R., Song X., Guo Y., Song P., Duan D., Chen Z.S. (2021). Natural Products: A Promising Therapeutics for Targeting Tumor Angiogenesis. Front. Oncol..

[22] Kwak E.A., Ahmed T., Flores P.C., Ortiz H.R., Langlais P.R., Mythreye K., Lee N.Y. (2023). Beta IV spectrin inhibits the metastatic growth of melanoma by suppressing VEGFR2-driven tumor angiogenesis. Cancer Med..

[23] Zhang P., Lai X., Zhu M.H., Shi J., Pan H., Huang Y., Guo R.J., Lu Q., Fang C., Zhao M. (2023). Jujuboside B suppresses angiogenesis and tumor growth via blocking VEGFR2 signaling pathway. Heliyon.

[24] Shah A.A., Kamal M.A., Akhtar S. (2021). Tumor Angiogenesis and VEGFR-2: Mechanism, Pathways and Current Biological Therapeutic Interventions. Curr. Drug Metab..

[25] Yang J., Wang R., Li H., Lv Q., Meng W., Yang X. (2016). Lentivirus mediated RNA interference of EMMPRIN (CD147) gene inhibits the proliferation, matrigel invasion and tumor formation of breast cancer cells. Cancer Biomark. Sect. A Dis. Markers.

[26] Pang Y., Wang K., Wang Y., Chenlin Z., Lei W., Zhang Y. (2018). Tumor-promoting and pro-angiogenic effects of roxarsone via VEGFR2/PLCgamma/PKC signaling. Chem. Biol. Interact..

[27] Wang Y., Yin D., Xu C., Wang K., Zheng L., Zhang Y. (2016). Roxarsone induces angiogenesis via PI3K/Akt signaling. Cell Biosci..

[28] Wang R., Sun S., Wang Z., Xu X., Jiang T., Liu H., Li X., Ren Z. (2022). MCPIP1 promotes cell proliferation, migration and angiogenesis of glioma via VEGFA-mediated ERK pathway. Exp. Cell Res..

[29] Yadav P., Dua C., Bajaj A. (2022). Advances in Engineered Biomaterials Targeting Angiogenesis and Cell Proliferation for Cancer Therapy. Chem. Rec..

[30] Lamalice L., Le Boeuf F., Huot J. (2007). Endothelial cell migration during angiogenesis. Circ. Res..

[31] Cominetti M.R., Terruggi C.H., Ramos O.H., Fox J.W., Mariano-Oliveira A., De Freitas M.S., Figueiredo C.C., Morandi V., Selistre-de-Araujo H.S. (2004). Alternagin-C, a disintegrin-like protein, induces vascular endothelial cell growth factor (VEGF) expression and endothelial cell proliferation in vitro. J. Biol. Chem..

[32] Nakashio A., Fujita N., Tsuruo T. (2002). Topotecan inhibits VEGF- and bFGF-induced vascular endothelial cell migration via downregulation of the PI3K-Akt signaling pathway. Int. J. Cancer.

[33] Benelli R., Albini A. (1999). In vitro models of angiogenesis: The use of Matrigel. Int. J. Biol. Markers.

[34] Norrby K. (2006). In vivo models of angiogenesis. J. Cell. Mol. Med..

[35] Troiani T., Schettino C., Martinelli E., Morgillo F., Tortora G., Ciardiello F. (2008). The use of xenograft models for the selection of cancer treatments with the EGFR as an example. Crit. Rev. Oncol./Hematol..

