# Supplementary figures and images for: A Recombinant Lentiviral Vegfr2-Silencing Vector Attenuates Roxarsone-Promoted Growth of Rat Vascular Endothelial Cells and Angiogenesis in Matrigel Plug and B16F10 Xenograft Models
Source: Vet Sci. 2024 Sep 24;11(10):451. doi: 10.3390/vetsci11100451 (PMC11511396; doi:10.3390/vetsci11100451)

Figure S1: Western blot image for VEGFR2.

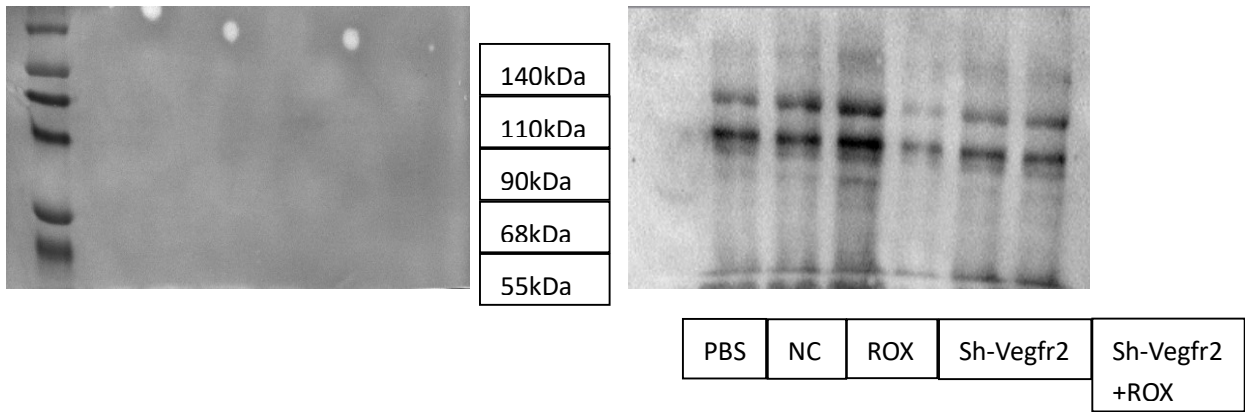

Supplement: Supplementary file 1 [file vetsci-11-00451-s001.zip › vetsci-3075921-supplementary.pdf]
